# The Ebola outbreak in West Africa: a story of related public health challenges and a pointer to solutions to mitigate the inevitable next outbreak

**DOI:** 10.11604/pamj.2014.19.48.5336

**Published:** 2014-09-22

**Authors:** Peter Nsubuga

**Affiliations:** 1 Global Public Health Solutions, Decatur, GA, USA

**Keywords:** Ebola, Public Health, Systems, Workforce

## Editorial

The current Ebola outbreak in West Africa has the world once again gripped by the threat of a zoonotic disease that has spread into several countries [1, 2]. While the underlying factors that have led to an increasing frequency of zoonotic disease outbreaks are complex, including population pressure and rapid movement between locations [3–5] in most of these epidemicsthere are least three related key drivers - a challenge of initial detection and appropriate investigation and response to a cluster of suspected cases, a challenge of management of an outbreak of a communicable disease, and a challenge of prediction or anticipation of what disease outbreak (both communicable and non-communicable) could come next (or is currently occurring but unseen). These three challenges reveal the urgent need of a public health workforce that is adequately trained and supported to operate multi-disease public health surveillance and response systems. Guinea, Sierra Leone, and Liberia do not yet have specific programs to develop this workforce and the system to support them while Nigeria does (and several other countries do) [6–8],and this difference is critical in addressing the major public heath challenge posed by the current Ebola outbreak as well as a myriad of communicable and non-communicable diseases. We will take a look at each of the three challenges in turn.

### The challenge of detecting and responding to the initial cluster of cases of an outbreak

Before a zoonotic disease outbreak occurs, there is often an outbreak in animals, whichserve as a reservoir for the disease, and some of these disease are endemic in the animals. Forsome species of the Ebola and Marburg filoviruses, researchers have found antibodies in several bat species [9]. Some researchers have also found evidence of Ebola infection in apes [10]. West Africa has a history of Lassa fever, which is carried by the multimammate ratMastomysnatalensis [11]. Staff who are adequately trained (meaning they have undergone experiential training) in field epidemiology, and are working in a multi-disease surveillance and response system that is supported adequatelywill know how to use case definitions to identify suspected cases of viral hemorrhagic fevers, and they will also know of the need to collect and transport specimens safely to a laboratory that can make the initial diagnosis. These staff will also know of the need and have the training to perform an outbreak investigation to confirm or rule out an outbreak, trace contacts, and initiate control measures to interrupt transmission of the suspected causative agent to the public. In several countries these field epidemiology staff have different but complimentary background training-either as medical, or veterinary, or laboratory scientists, each of them providing critical comparative advantages as they operate multi-disease surveillance and response systems [6]. Most countries have guidelines and documents that define what to do for public health surveillance and response [12], but the countries that are able to perform well are the ones that have invested (or been supported to invest) in developing a competently trained public health workforce and developed the systems to support them [13]. In effect, having documents alone without competently trained public health workers to make the decisions that will save lives is a waste. Missing or mishandling the initial cluster of cases often leads to an unpredictable number of cases that can quickly overwhelm a country's public health and economic infrastructure as we are unfortunately currently witnessing. There have been estimates of several thousands of cases that will occur before this outbreak is controlled, and some authors have modeled varying scenarios of the Ebola reproductive rate (R0) from previous outbreaks which are illustrative'an R0 at 2.7 (95% CI 1.9-2.8) for the 1995 DRC epidemic and at 2.7 (95% CI 2.5-4.1) for the 2000 Uganda outbreak [14], meaning each Ebola case could lead to up to an average of 3 secondary cases. The economic cost of a raging outbreak is far greater than the cost to develop a competently trained public health workforce and implement multi-disease surveillance and response systems for them to work in.

### The challenge of managing an outbreak of a communicable disease

Outbreak management adds to outbreak investigation in several important ways, principally in whereas outbreak investigation and confirmation are primarily carried out by staff trained in field epidemiology, outbreak management is a multi-sectoral, multi-skill effort, which increases in complexity with larger outbreaks but revolves around at least fivegroups of core activities. These activities are: a) epidemiology and surveillance to characterize the outbreak, identify and trace contacts, and determine where the outbreak is headed and how it is transmitted, typically done by field epidemiologists; b) case management and infection control, to humanely treat and isolate cases while performing infection control, and safely burying any dead patients; c) laboratory activities which include testing specimens from suspected cases, clinical tests during treatment, biosafety, and specimen transfers for more specialized tests; d) community activities which include health education of all susceptible groups in the general and special population groups (which include health workers) about how to interrupt transmission and avoid becoming cases--the community activities are critical because they should build trust with the affected people and enable them to cooperate to identify suspected cases humanely; and e) activities in the environment to determine where the reservoir and vectors of the disease are and how to address them. These five activities are interconnected by a coordination function, which leads to decisions on logistical needs, regulations to enforce, and vaccinations and chemoprophylaxis if necessary. The coordination function also includes frequent, clear and transparentcommunication with the public, the media, and authorities to build a sense of trust, effectively a “we are in it together” common purpose to confront and end the outbreak [15]. Whereas the workforce that is needed in outbreak management is multi-sectoral, ultimately the best decisions are guided by the data that come from competently trained field epidemiologists who have differing background training. Several countries coordinate large outbreaks in an emergency operations center, which allows for a focused effort away from the usual ministry of health work [16].

### The challenge of predicting the next outbreak in a country

The current Ebola outbreak suggests a challenge with predicting the occurrence of zoonotic disease outbreaks that can be mitigated with collaboration between the veterinary and public health sectors on zoonoses. Zoonotic disease outbreaks will continue to occur; current data indicate that >60% of emerging or re-emerging diseases are zoonotic, affecting people who have no immunity to these diseases [4, 5]. Along with zoonoses there are several disease outbreaks that have spread with in and beyond country borders, for example West and Central Africa had a major cholera epidemic recently [17, 18]. Additionally non-communicable diseases are at epidemic levels even if they do not provide the same level of alarm. A look at the level of hemorrhagic strokes caused by untreated hypertension [19] or the rising levels of obesity [20], or the unattended epidemic of mental illness makes that point [21]. However in the case of communicable diseases particularly zoonoses, there is an opportunity to combine veterinary surveillance with public health surveillance to identify diseases before they cross the “border” from animals to humans. Joint training of veterinarians and health workers (physicians, nurses, and laboratory scientists) in field epidemiology and laboratory training programs achieves the workforce that is needed to implement prediction-based surveillance and response systems for zoonotic diseases [8]. New initiatives that allow the public health and veterinary sectors to combine surveillance data using electronic methods (e.g., One Health Surveillance Initiative) will likely facilitate prediction-based surveillance [22]. In summary the current Ebola outbreak in West Africa reveals three related public health systems and workforce challenges that need to be addressed in order for Africa to be able to address communicable and non-communicable disease threats. A locally developed public health workforce that comprises medical and non-medical personnel who are competently trained in field epidemiology and is supported in multi-disease surveillance and response systems best addresses these challenges. Acting now in each country large or small to implement or continue to support these staff and systems will be cheaper than acting later, as surely these staff and systems will be needed at some point in each country.
